# Alvopem^®^ (pemetrexed) safety assessment in patients with non-small cell lung cancer or malignant pleural mesothelioma: a post-marketing surveillance

**DOI:** 10.1186/s40545-023-00524-5

**Published:** 2023-01-25

**Authors:** Sharareh Seifi, Babak Salimi, Zahra Esfahani Monfared, Cyrus Sabahi, Hamidreza Kafi, Adnan Khosravi

**Affiliations:** 1 Chronic Respiratory Disease Research Center, National Research Institute of Tuberculosis and Lung Diseases (NRITLD), Shahid Beheshti University of Medical Sciences, Tehran, Iran; 2 Faculty of Pharmacy, Tehran University of Medical Sciences, Tehran, Iran; 3 Medical Department, Orchid Pharmed Company, No 12, 70th Alley, Seyed Jamaledin Asad Abadi St., Yousef Abad, Tehran, Iran; 4 Masih Daneshvari Hospital, Darabad Avenue, Shahid Bahonar Roundabout, Tehran, 1956944413 Iran

**Keywords:** Non-small cell lung cancer, Observational study, Pemetrexed, Post-marketing surveillance, Safety

## Abstract

**Background:**

Lung cancer is the leading cause of cancer deaths worldwide in both men and women, and non-small cell lung cancer (NSCLC) accounts for the majority (~ 85%) of lung cancers. This post-marketing surveillance (PMS) study aimed to evaluate the safety of Pemetrexed (Alvopem^®^, NanoAlvand, Iran) in Iranian patients with lung cancer or mesothelioma.

**Methods:**

The present study is an observational, single-center, open-label, and post-authorization study. All eligible non-squamous NSCLC and malignant pleural mesothelioma (MPM) patients who received pemetrexed based on the physicians’ decision, were enrolled.

**Results:**

A total of 199 patients with non-squamous NSCLC [186 patients (93.47%) or MPM (12 patients (6.03%)] were enrolled from March 2016 to February 2020. The most common reported adverse event (AE) was anemia (89.39%), followed by neutropenia (28.79%) and leukopenia (24.75%). The most important grade 3 AEs were anemia and neutropenia, with the incidence rate of 3.54% and 7.58%, respectively. No grade 4 AEs were reported. Moreover, the results of our study showed negative statistically significant correlations between patients’ age and mean neutrophil count (*r* = − 0.17; *P* = 0.0156) and hemoglobin (*r* = − 0.16; *P* = 0.0201) in all six visits.

**Conclusions:**

The results of this open-label, observational PMS showed that Pemetrexed (Alvopem^®^) is safe in patients with non-squamous NSCLC patients receiving pemetrexed-containing regimens.

*Trial registration*: The trial was registered at ClinicalTrials.gov (NCT04843007) in April 13th, 2021.

## Background

Lung cancer is the leading cause of cancer deaths worldwide in both men and women. Based on the international Agency for research on cancer report, the 5-year prevalence of all cancer sites and lung cancer In Iran is 319,740 and 11,703, respectively. In 2022, a review article conducted in Iran showed lung cancer incidence was mainly in the sixth decade of a patient’s life [1, 2]. Non-small cell lung cancer (NSCLC) accounts for the majority (~ 85%) of lung cancers, with the remainder as mostly small cell lung cancer (SCLC) [3]. Malignant pleural mesothelioma (MPM) is an aggressive and rare cancer of the membrane covering the lungs and the inner side of the ribs which is mostly caused by inhaling asbestos [4]. Reports that discussed the correlation between advanced age and the incidence of these two cancers revealed that more than 50% of lung cancer patients and most MPM patients were diagnosed in the senior adult age group [5–7].

Pemetrexed is an anti-folate medicine used in non-squamous non-small cell lung cancer patients or malignant pleural mesothelioma patients who are not candidates for curative surgery. It disturbs folate-dependent processes necessary for cell replication by inhibiting enzymes synthesizing purine and thymidine nucleotide and protein [8].

In 2003, a phase 3 clinical study evaluated the efficacy of pemetrexed in patients with MPM when added to cisplatin. Chemotherapy-naive patients who were not eligible for curative surgery were randomly assigned to receive pemetrexed 500 mg/m^2^ and cisplatin 75 mg/m^2^ on day 1, or cisplatin 75 mg/m^2^ on day 1. The results showed that the survival, mortality rate, and response to treatment were in favor of the pemetrexed group [9]. According to these findings, the United States Food and Drug Administration (U.S. FDA) approved pemetrexed for treatment of MPM in combination with cisplatin for patients whose disease is either unresectable or who are not otherwise candidates for curative surgery in February 2004 [10, 11]. In 2011, a study was performed on patients with recurrent MPM after treatment with pemetrexed. Re-treatment with pemetrexed could increase overall survival (OS) for 10.5 months. Toxicity was mild, and grade 3 or 4 hematological toxicity occurred in 9.7% of patients [12]. According to ESMO Clinical Practice Guidelines, Pemetrexed-containing regimens is a first-line treatment option for unresectable MPM patients [13].

Another phase 3 study was conducted to evaluate the therapeutic effect of cisplatin plus gemcitabine and cisplatin plus pemetrexed in chemotherapy-naïve NSCLC patients. In advanced NSCLC, cisplatin/pemetrexed provided similar efficacy with better tolerability and more convenient administration than cisplatin/gemcitabine [14]. In September 2008, the FDA granted pemetrexed approval as a first-line treatment, in combination with cisplatin, against locally advanced and metastatic non-squamous NSCLC [15–17].

A systematic meta-analysis was conducted to compare the efficacy and safety of pemetrexed and docetaxel for NSCLC in 2014. Six randomized clinical trials (RCTs) involving 1414 patients were identified. There were no statistically significant differences in overall response rate (ORR), survival time, progression-free survival (PFS), disease control rate, and 1–2-year survival rates (*P* > 0.050), but it is worthy of mentioning that patients in the pemetrexed arm had significantly higher 3-year survival rate (*P* = 0.002). As for safety, pemetrexed led to lower rates of grade 3–4 febrile neutropenia, neutropenia, leukocytosis, diarrhea and alopecia. However, it was associated with a higher rate of grade 3–4 thrombocytopenia [18].

In 2014 another study reviewed the safety and effectiveness of pemetrexed in patients with NSCLC using data from post-marketing surveillance (PMS). Among 699 patients registered in Japan from June 2009 to May 2010, 683 patients were analyzed (343: first-line therapy, 340: second-line therapy or beyond). The surveillance results showed no apparent difference in total adverse drug reactions (ADRs) compared to the safety profile established in clinical trials previously conducted in Japan and overseas. These results demonstrated the safety and effectiveness of pemetrexed treatment for NSCLC patients in daily clinical settings [19]. According to results of a phase 3 study that added pemetrexed to bevacizumab for maintenance therapy, in the combination arm, the OS was improved but not statistically significant (23.3 vs. 19.6 months, *P* = 0.069), although the PFS improved significantly (5.7 vs. 4.0 months, *P* < 0.001). The safety profile was similar to previous studies [20].

Also, in a systematic review and meta-analysis in 2016, the efficacy of pemetrexed plus platinum doublet chemotherapy for advanced non-squamous NSCLC was evaluated. Ten trials involving 2551 patients were identified. The results showed that adding pemetrexed to platinum doublet chemotherapy improves OS significantly compared to other platinum-based chemotherapies [21].

The generic version of pemetrexed (Alvopem^®^, NanoAlvand, Iran) has been widely administered in Iran since its marketing authorization in 2016 [22]. Post-marketing surveillance (PMS) is a method for evaluating drug safety and effectiveness in the real-world setting. Thus, in this post-marketing, observational study, we aimed to investigate the safety of pemetrexed in patients with NSCLC and MPM.

## Methods

### Design and treatment

The present study was an observational, single-center, open-label, and PMS study that evaluated the safety of pemetrexed in Iran. All data were recorded by the designated physicians in a booklet containing information on chemotherapy (induction therapy with pemetrexed up to 6 cycles). Patients' demographic data, habitual history, cancer types and stages, and other chemotherapy medications were provided at the baseline visit. In addition, patients’ laboratory tests, pemetrexed information and administration protocol checklist, premedication regimen, adverse drug events, para-clinical actions due to an adverse event (AE), and clinical actions due to AE were recorded for all the participants in each visit. Reported adverse events were assessed by physicians and assessment was conducted based on WHO–UMC system for causality assessment. Designated physicians decided on the administered dose and the duration of therapies.

Ethics approval was obtained from the Institutional Research Ethics Committee of the principal investigator’s affiliated university (Beheshti University of Medical Sciences; IR.SBMU.NRITLD.REC.1398.061) in October 22th, 2019. The study was carried out according to the principles of the Declaration of Helsinki. All the participants voluntarily signed a written informed consent prior to participation in the study. The trial was registered at ClinicalTrials.gov (NCT04843007).

### Patients

Since this was an open-label non-interventional PMS, all eligible non-squamous NSCLC and MPM patients who received Pemetrexed (Alvopem^®^) based on the physician’s decision were enrolled in this study. The study was conducted in the chronic respiratory disease center at the National Research Institute of Tuberculosis and Lung Disease (NRITLD), Iran.

### Objective

The objective of the current study was safety assessment, including the incidence of AEs and serious adverse events (SAEs). The intensity of AEs was determined based on the Common Terminology Criteria for Adverse Events version 5.0 (CTCAE v5.0). The terminology for AEs was chosen using the Medical Dictionary for Regulatory Activities (MedDRA) (MedDRA Desktop Browser 4.0 Beta) system organ class (SOC) and preferred term (PT). The causality of AEs was assessed based on the World health organization–Uppsala Monitoring Centre (WHO–UMC) system for standardized case causality assessment.

### Statistical analysis

In a cohort study with no background incidence of a rare adverse reaction (interstitial), by assuming 80% power, a 5% level of significance, and a 5% dropout rate, the required sample size was 200 [23]. Demographic data and baseline characteristics were analyzed using descriptive statistics. Summary statistics included mean and standard deviation for continuous variables and frequency and percentage for categorical variables. Based on the definition of incidence, the number of patients with at least one new AE was counted. Moreover, the frequency of adverse events based on the causal relationship was assessed, and the incidence of at least possibly related AEs were reported. All data cleaning procedures and analyses were performed using STATA 14 (Stata Corp LP, College Station, USA).

## Results

### Patients

A total of 199 patients with non-squamous NSCLC or MPM were enrolled in this PMS study from March 2016 to February 2020. One patient was excluded from the analyses as the diagnosis was not recorded. Patients received a mean number of 4.6 cycles of chemotherapy regimen containing pemetrexed with the dose of 500 mg/m^2^ and carboplatin with the dose of Area Under Curve 5 (AUC5). In addition, all patients received recommended premedication according to the related guidelines. Patients’ baseline characteristics are summarized in Table 1.

**Table 1:** Patients’ baseline characteristics

Variable	Value (*N* = 198)
Age (y), mean ± SD	61.11 ± 11.36
Weight (kg), mean ± SD	66.93 ± 14.74
Height (cm), mean ± SD	166.44 ± 9.88
BSA (m^2^), mean ± SD	1.75 ± 0.21
Smokers, *n* (%)	132 (66.67)
Diagnosis, *n* (%)	
NSCLC	186 (93.94)
MPM	12 (6.06)
Sex, *n* (%)	
Male	130 (65.66)
Female	68 (34.34)
Stage 4, *n* (%)	
NSCLC	168 (90.32)
MPM	2 (16.67)
Laboratory tests, mean ± SD	
SCr (mg/dL)	1.02 ± 0.22
Bilirubin (mg/dL)	0.46 ± 0.30
BUN (mg/dL)	31.24 ± 10.34
AST (units/L)	23.78 ± 21.72
ALT (units/L)	23.99 ± 17.83
PLT (cells/μL)	324,653 ± 109,183
WBC (cells/μL)	10,519 ± 4920
Neutrophil (cells/μL)	7743 ± 4541
Hb (g/dL)	12.68 ± 1.90

*SD* standard deviation, *BSA* body surface area, *NSCLC* non-small cell lung cancer, *MPM* malignant pleural mesothelioma, *SCr* serum creatinine, *AST *aspartate aminotransferase, *ALT* alanine transaminase, *PLT* platelet, *WBC* white blood cell, *BUN* blood urea nitrogen, *Hb* hemoglobin

### Safety analysis

During this study, the most common reported AEs were anemia (89.99%) followed by neutropenia (29.29%) and leukopenia (24.75%) among individuals treated with pemetrexed. The incidence of all AEs and the incidence of grade 3 and 4 of AEs are presented in Table 2 based on SOC and PT. The gender-based analysis of the hematologic adverse events are provided in Table 3.

**Table 2:** Incidence of AEs classified by SOC and PT

System organ class	Preferred term	All grades *N* (%)	Grade 3 and 4 *N* (%)
Blood and lymphatic system disorders	Anemia	178 (89.99)	15 (7.58)
	Leukopenia	49 (24.75)	0 (0)
	Neutropenia	58 (29.29)	7 (3.54)
	Thrombocytopenia	20 (10.10)	0 (0)
Investigations	Alanine aminotransferase increased	20 (10.10)	0 (0)
	Aspartate aminotransferase increased	12 (6.06)	1 (0.51)
	Creatinine renal clearance decreased	48 (24.24)	0 (0)
Renal and urinary disorders	Renal disorder	1 (0.51)	0 (0)
General disorders and administration site conditions	Drug intolerance	1 (0.51)	0 (0)
No. of Patients with At Least one AE		190 (95.96)	22 (11.11)

*AE* adverse event

**Table 3:** Incidence of hematologic AEs classified by SOC and PT based on gender

System organ class	Preferred term	Male *N* (%)	Female *N* (%)
Blood and lymphatic system disorders	Anemia	121 (93.07)	57 (83.82)
	Leukopenia	29 (22.31)	20 (29.42)
	Neutropenia	32 (24.61)	26 (38.24)
	Thrombocytopenia	13 (10)	7 (10.29)

The causality of AEs among 198 patients is shown in Table 4. ‘Possible’ was the most common causality assessed in this study (82.13%).

**Table 4:** Frequency of AEs based on causality assessment

Causality	*N* (%)
Possible	786 (82.13)
Unlikely	141 (14.73)
Unassessable/unclassifiable	30 (3.13)
Total	957 (100)

*AE* adverse event

The number of patients who experienced at least one possibly related AE is 184 (92.93%). In the current study, among 198 patients, 70 patients (35.35%) (23 men and 47 women) experienced at least one SAE. A total of 45 (22.73%) at least possibly related SAEs were reported.

### Exploratory analysis

Subgroup analysis was performed using the Chi-square test to compare the incidence of anemia in different age categories. The findings showed that in patients aged greater or lower than 56, the incidence of anemia is statistically different (92.48% vs. 83.08%; *P* = 0.044).

The Pearson correlation coefficient was used to determine if an association existed between the patients’ age and mean neutrophil counts, and mean hemoglobin levels. There were a weak but statistically significant negative correlations between patients’ age, and mean neutrophil counts (*r* = − 0.17; *P* = 0.016) and Hb (*r* = − 0.16; *P* = 0.020) in all cycles of treatment.

## Discussion

Real-world studies are of significant importance in the clarification of the medicines’ safety profile in the post-authorization period. The current PMS was designed to evaluate the safety of a generic version of pemetrexed (Alvopem^®^, NanoAlvand, Iran) in a real-world clinical setting in NSCLC and MPM patients in Iran.

Giorgio V. Scagliotti et al. studied pemetrexed-based regimens in chemo-naive patients with locally advanced or metastatic NSCLC [24]. Patients were randomly assigned to receive pemetrexed 500 mg/m^2^ plus oxaliplatin 120 mg/m^2^ (PemOx) or pemetrexed plus carboplatin AUC 6 (PemCb). The rates of grade 3/4 neutropenia and anemia were 25.7% and 7.7%, respectively. In another phase II study, Radj Gervais et al. evaluated pemetrexed and carboplatin as an active option in the first-line treatment of elderly patients with advanced NSCLC. Based on their findings, 51.6%, 30.7%, and 19.4% of grade 3/4 neutropenia, leukopenia, and anemia were reported, respectively [25]. In a review article in 2010, Fuld et al. addressed several studies on pemetrexed as second-line, first-line, and maintenance therapy in NSCLC. Some early studies demonstrated the important role of B12 and folate supplementation marked by significant grade 3/4 myelosuppression. This review reported the incidence rates of 21–40% for grade 3/4 neutropenia [26].

In a study on patients with advanced NSCLC who received pemetrexed or pemetrexed and carboplatin as the second-line chemotherapy, Andrea Ardizzoni et al. recorded 11.6%, 8%, and 5.4% of grade 3/4 neutropenia, leukopenia, and anemia, respectively [27].

In another study on the efficacy and safety of pemetrexed plus cisplatin as first-line chemotherapy in advanced malignant peritoneal mesothelioma, Nagata et al. reported among hematological toxicities, Incidence of grade 3/4 leukopenia, neutropenia, anemia and thrombocytopenia were 21%, 17%, 14% and 3%, respectively [28]. Moreover, in a study for assessment of efficacy and safety of pemetrexed maintenance chemotherapy for advanced NSCLC in a real-world setting conducted in 2021, authors have mentioned grade 1/2 hematological toxicities as the most commonly reported toxicities with leukopenia in 35.2% and neutropenia in 23.9% of patients [29].

In the current PMS, no grade 4 AEs or interstitial lung disease (ILD) were reported. The most important grade 3 AEs were neutropenia and anemia, with the incidence of 3.54% and 7.58%, respectively. Our findings are generally in line with Ardizzoni et al. study. The rates of AEs in our study and Ardizzoni et al. study are relatively lower compared to the above-mentioned studies. These findings are consistent with our former study on another generic product of pemetrexed in Iranian patients with advanced NSCLC [30]. Furthermore, we found a negative correlation between age and neutrophil count or Hb.

The results regarding the subgroup analysis of anemia in different age groups and the negative correlation between age and neutrophil count or hemoglobin levels show that the incidence of anemia is higher in patients older than 56. Moreover, it seems that the chance of neutropenia will also increase with increasing age.

This study had some limitations, including the low number of patients with MPM, which makes the generalizability of the safety results to this population difficult. Moreover, the lack of a control group must be taken in to account while interpreting the results.

## Conclusions

The results of this open-label, observational PMS study showed that the generic version of pemetrexed (Alvopem^®^) is safe in patients with non-squamous NSCLC receiving pemetrexed-containing regimens.

## Data Availability

The data sets generated during and/or analyzed during the current study are available from the corresponding author on reasonable request.

## Abbreviations

NSCLC: Non-small cell lung cancer
PMS: Post-marketing surveillance
MPM: Malignant pleural mesothelioma
AE: Adverse event
SCLC: Small cell lung cancer
U.S. FDA: United States Food and Drug Administration
OS: Overall survival
ORR: Overall response rate
PFS: Progression-free survival
ADR: Adverse drug reactions
NRITLD: National Research Institute of Tuberculosis and Lung Disease
SAE: Serious adverse event
CTCAE v5.0: Common Terminology Criteria for Adverse Events version 5.0
MedDRA: Medical Dictionary for Regulatory Activities
SOC: System organ class
PT: Preferred term
WHO–UMC: World health organization–Uppsala Monitoring Centre
BSA: Body surface area
AUC: Area under curve
ILD: Interstitial lung disease
PemOx: Pemetrexed 500 mg/m^2^ plus oxaliplatin 120 mg/m^2^
PemCb: Pemetrexed plus carboplatin AUC 6
RCT: Randomized clinical trial
SD: Standard deviation
SCr: Serum creatinine
BUN: Blood urea nitrogen
AST: Aspartate aminotransferase
ALT: Alanine transaminase
Plt: Platelet
WBC: White blood cell
Hb: Hemoglobin
